# Analysis of Autophagy-Related Signatures Identified Two Distinct Subtypes for Evaluating the Tumor Immune Microenvironment and Predicting Prognosis in Ovarian Cancer

**DOI:** 10.3389/fonc.2021.616133

**Published:** 2021-05-10

**Authors:** Xingyu Chen, Hua Lan, Dong He, Zhanwang Wang, Runshi Xu, Jing Yuan, Mengqing Xiao, Yao Zhang, Lian Gong, Songshu Xiao, Ke Cao

**Affiliations:** ^1^ Department of Oncology, Third Xiangya Hospital of Central South University, Changsha, China; ^2^ Department of Obstetrics and Gynecology, Third Xiangya Hospital of Central South University, Changsha, China; ^3^ The Second People’s Hospital of Hunan Province, Hunan University of Chinese Medicine, Changsha, China; ^4^ Medical School, Hunan University of Chinese Medicine, Changsha, China

**Keywords:** ovarian cancer, prognostic risk signature, autophagy-related genes, tumor immune microenvironment, immunotherapy

## Abstract

Ovarian cancer (OC) is one of the most lethal gynecologic malignant tumors. The interaction between autophagy and the tumor immune microenvironment has clinical importance. Hence, it is necessary to explore reliable biomarkers associated with autophagy-related genes (ARGs) for risk stratification in OC. Here, we obtained ARGs from the MSigDB database and downloaded the expression profile of OC from TCGA database. The k-means unsupervised clustering method was used for clustering, and two subclasses of OC (cluster A and cluster B) were identified. SsGSEA method was used to quantify the levels of infiltration of 24 subtypes of immune cells. Metascape and GSEA were performed to reveal the differential gene enrichment in signaling pathways and cellular processes of the subtypes. We found that patients in cluster A were significantly associated with higher immune infiltration and immune-associated signaling pathways. Then, we established a risk model by LASSO Cox regression. ROC analysis and Kaplan-Meier analysis were applied for evaluating the efficiency of the risk signature, patients with low-risk got better outcomes than those with high-risk in overall survival. Finally, ULK2 and GABARAPL1 expression was further validated in clinical samples. In conclusion, Our study constructed an autophagy-related prognostic indicator, and identified two promising targets in OC.

## Introduction

Ovarian cancer (OC) is one of the most lethal gynecologic malignant tumors (1). Due to the nonspecific symptoms in the early stage and the lack of effective screening techniques of the disease, a large number of patients are diagnosed at an advanced stage, of which the 5-year survival rate was less than 30%. Cytoreductive surgery and platinum- and paclitaxel-based chemotherapy are still the basic treatments for OC. Despite advances in combination chemotherapy, targeted therapy and intraperitoneal chemotherapy, 80% of OC patients initially respond to treatment, but most eventually relapse and ultimately develop into a chemotherapy-resistant disease; thus, no significant improvement in the prognosis of OC has been achieved (2–4).

The clinicopathological features of OC are predicted by the WHO classification and TNM staging system of tumor lymph node metastasis, which is also the key for selecting appropriate treatment. However, because of the heterogeneity of OC, there are obvious stratifications into histological or molecular subtypes, and the results may be significantly different even for patients with similar clinical features and treatment regimens. These observations showed that the clinicopathological features and current classification are not sufficient for prediction and risk stratification. Consequently, it is difficult to meet the needs of clinicians (5, 6). Therefore, it is of great significance for improving the prognosis of OC to search for specific prognostic biomarkers and therapeutic targets with higher predictive value.

Tumor immunotherapy has become a promising treatment strategy, which aims to restore the immune response to fight against tumors. Immunotherapies such as immune checkpoint therapy (ICT), tumor vaccines, immune adoptive therapy and immunomodulators have been applied in many cancers. Many immunosuppressive receptors have been identified and studied in tumors; these studies have led to the development of therapies including, but not limited to, FDA-approved monoclonal antibodies that mediate clinically relevant immunostimulatory effects by suppressing immunosuppressive receptors, such as PD-L1, PD-1, CTLA-4, LAG3, TIGIT and BTLA (7–9). The application of immunotherapy has significantly changed the strategies and modes of treating OC and greatly improved the quality of life in some patients with OC. Pembrolizumab, nivolumab and avirumumab are anti-PD-1 or anti-PD-L1 monoclonal antibodies, and bevacizumab is a monoclonal antibody that binds to vascular endothelial growth factor (VEGF). These drugs have been successfully used to treat recurrent or drug-resistant OC (10–12). However, only some patients benefit from immune treatment, and some patients still show poor responses or resistance to immunotherapy. How to successfully identify which patients may benefit from immunotherapy and which patients may exhibit poor responses or resistance to immunotherapy is a clinically difficult problem. Therefore, screening subjects suitable for immunotherapy would help increase the success rate of treatment and benefit more patients.

Autophagy is an important immunomodulatory process in the tumor microenvironment that can maintain the homeostasis, activation and biological function of immune cells. Innate immune-mediated autophagy can be upregulated by activating innate immune receptors, including Toll-like receptors (TLRs) and nucleotide oligomeric domain (NOD)-like receptors (NLRs) (13). The adaptive immune response depends on the recognition of extracellular or intracellular peptide epitopes provided by major histocompatibility complex II (MHCII) and MHCI molecules and recognized by CD4^+^ T and CD8^+^ T cells, respectively. Autophagy provides ATP molecules for antitumor T cells to activate antigen-presenting cells (APCs) (14). Autophagy also plays a role in protecting cells and tissues from stress in normal physiological processes. However, inappropriate autophagy may lead to low antitumor immunity, affect infiltrating of immune cells, and inhibit the immune response to weaken immunotherapy (15). Recent studies have shown that autophagy is closely related to tumor immunotherapy and clinical prognosis of OC (16). Targeted autophagy may be a promising therapeutic strategy for improving the efficacy of immunotherapy and enhancing the immune response. However, it is still difficult to find effective and appropriate autophagy-related gene biomarkers to identify and evaluate tumor-specific cellular immune responses in tumor patients and to predict patient responses to immunotherapy.

In our study, we downloaded the expression profile of OC patients from the TCGA database and obtained autophagy-related genes (ARGs) from the MsigDB database. Based on the ARGs, OC patients were successfully classified into two subtypes, and a risk model was established to assess the prognosis of OC. The system comprehensively evaluated the correlation among classification, risk model and TIM and explored the effect of autophagy on the regulation of the OC TIM. ARGs may be potential biomarkers and provide new ideas for immunotherapy.

## Materials and Methods

### Datasets and Samples

The gene expression profile was experimentally measured using the Illumina HiSeq 2000 RNA Sequencing platform by the University of North Carolina The Cancer Genome Atlas (TCGA, https://portal.gdc.cancer.gov/) genome characterization center. Level 3 data were downloaded from the TCGA data coordination center,307 ovarian cancer samples were included. This dataset shows the gene-level transcription estimates, as in log2(x+1) transformed RSEM normalized count. The genes are mapped onto the human genome coordinates using HUGO probeMap, refering to method description from University of North Carolina TCGA genome characterization center. For all the cohorts, only patients with available expression profiles, clinicopathological (including age, status, lymphovascular invasion, stage) data and survival data were included in the analyses. The eight OC samples and paired adjacent tissue samples were obtained from Third Xiangya Hospital of Central South University.

### Identification of OC Subtypes

We obtained the autophagy gene set from the MSigDB (https://www.gsea-msigdb.org/gsea/msigdb/search.jsp) KEGG_REGULATION of AUTOPHAGY of molecular signatures; we obtained a total of 35 autophagy-related genes. Compared with the expression data of the genes in the TCGA, candidate genes whose expression level was too low (log2(x+1) <1) were excluded, and 19 ARGs were obtained. We performed k-means clustering based on the mRNA expression data of 19 autophagy-related genes (17). Before performing k-means, a filtering procedure was conducted. We performed k-means (“kmeans” function in R) clustering and used the “NbClust”, “cluster” and “factoextra” packages in R to determine the optimal number of OC subtypes. The values of k where the magnitude of the cophenetic correlation coefficient began to fall were chosen as the optimal number of clusters.

### Estimation of Immune Infiltration

The inference of infiltrating cells in the tumor microenvironment (TME) was used to quantify the level of immune cell infiltration in the OC samples. Based on the immune cell marker genes provided by Bindea G, et al (18), we used the R language GSVA package and then used single-sample gene set enrichment analysis (ssGSEA) to quantify the levels of infiltration of 24 immune cells, including T lymphocytes, dendritic cells, natural killer cells, etc., into the sample based on the immune cell marker gene expression profile data in the TCGA-OV. According to the level of immune cell infiltration, we divided patients into a high-infiltration group and a low-infiltration group, observed the relationship between age, stage, grade, lymphatic metastasis and immune infiltration, and used a heat map display to observe various immune cells in the high- and low-risk groups. Finally, the Pearson correlation coefficient was used to calculate the correlation coefficient among immune cells, risk genes and immune cells.

### Screening of Differentially Expressed Genes (DEGs) and Bioinformatics Analysis

To obtain the DEGs between cluster A and cluster B in the TCGA–OV cohort, the R package “limma” was used in the standard comparison mode. The DEG threshold was set to | logFC | > 1, and the significance criteria for identifying DEGs was set as an adjusted P value <0.05. Metascape (http://metascape.org) is an online interactive website that helps biologists perform functional enrichment analyses on specific gene sets (19). We first introduced the 19 ARGs into Metascape and identified all the statistically enriched terms. The remaining significant terms were then hierarchically clustered into a tree based on κ-statistical similarities among their gene memberships. Then, we analyzed all the transcripts based on the fold change (log2) obtained by difference analysis of the two different OC subtypes using Gene Set Enrichment Analysis (20) (GSEA, http://software.Broadstitute.org/GSEA/) to evaluate the skewness of the two distributions of the selected genes in the list of ranked genes. Then we set the GSEA threshold for significantly enriched functional annotations to P value<0.05 and | Normalized Enrichment Score (NES) |≥1.

### Identification and Validation of the Prognostic Gene Signature

The “glmnet” R package was utilized to carry out the LASSO COX regression. The “glmnet” R package was utilized to carry out the univariate Cox regression to screen 19 ARGs. Using the “glmnet” software package of R for LASSO Cox regression analysis, seven autophagy-related genes were screened to determine the best predictive model, and these genes were selected to further calculate the risk score of each patient (21, 22):

$$\text{riskscore}={\text{Expression}}_{\text{mRNA}1}\times {\text{Coefficient}}_{\text{mRNA}1}+{\text{Expression}}_{\text{mRNA}2}\times \;{\text{Coefficient}}_{\text{mRNA}2}+\ldots {\text{Expression}}_{\text{mRNAn}}\times {\text{Coefficient}}_{\text{mRNAn}}$$

According to the median risk coefficient, the patients are divided into a high-risk group and a low-risk group. The Cox proportional hazard regression model includes risk score, age, grade, lymphatic invasion, and staging. The hazard ratio (HR) value distinguishes the prognostic predictors of risk genes and protective genes (HR>1 is a risk gene, HR<1 is a protective gene, p<0.05). Subsequently, Kaplan-Meier survival analysis was performed, and the sensitivity and specificity of the ROC curve were used to evaluate the prognostic performance of the signature. Circos is a visualization tool that can be used to identify and analyze the similarities and differences generated by genome comparisons, effectively display changes in genome structures, and generally display any other types of positional relationships between genome intervals (23, 24). The mutations of 7 ARGs in the TCGA-OV cohort were downloaded from the cbioportal website (https://www.cbioportal.org/). We used Pearson correlation analysis to analyze the correlation between the risk score and common immune checkpoints and tried to use the risk score to accurately predict the effect of treatment.

### Quantitative Real-Time PCR (qRT-PCR)

Total RNA was isolated from the OC samples using TRIzol reagent (Invitrogen). The PrimeScript RT (reverse transcription) Kit (TaKaRa Bio) was used to obtain cDNA. qRT-PCR was conducted using the AceQ qPCR SYBR Green Master Mix (Vazyme). β-actin was used as internal control.

**Table:** 

Primer	Sequence (5′–3′)
ULK2-F	TTTGGTGCCACACAACATCT
ULK2-R	GGAACTGGAATTGGTGCTGT
GABARAPL1-F	ATGAAGTTCCAGTACAAGGAGGA
GABARAPL1-R	GCTTTTGGAGCCTTCTCTACAAT
β-actin-F	ACCCTGAAGTACCCCATCGAG
β-actin-R	AGCACAGCCTGGATAGCAAC

### Western Blotting (WB)

Tissue samples were lysed using RIPA buffer in the presence of Protease Inhibitor Mixture and PhoSTOP (Roche Applied Science). The protein concentration was quantified using a bicinchoninic acid protein assay kit (Thermo Fisher Scientific). Subsequently, the protein (30 μg) was separated by 10% SDS-PAGE and transferred onto polyvinylidene fluoride membranes (Millipore). The membranes were then blocked with 5% nonfat milk in TBS and incubated at 4°C overnight with the following primary antibodies: anti-ULK2 (dilution 1:4000, Omnimabs, OM294638); anti-GABARAPL1 (dilution 1:4000, Abcam, ab86497) and β-actin (dilution 1:4000, Ptgcn, 66009-1-Ig). Then, the membranes were incubated with goat anti-rabbit IgG/HRP secondary antibodies and washed. Finally, the bands were visualized using enhanced chemiluminescence.

### Immunohistochemistry (IHC)

Tissues were derived from clinical specimens. The tissue sections were deparaffinized, hydrated, repaired and blocked with citric acid antigen. Subsequently, the tissue sections were probed with an ULK2 antibody (1:50, Omnimabs, OM294638) and GABARAPL1 antibody (1:200, Abcam, ab86497) at 4°C overnight. The sections were washed with PBS for 5 times, and then a secondary antibody was added and incubated at room temperature for 10 minutes. DAB and hematoxylin were added for visualization.

### Statistical Analysis

All of the analyses were performed with R software (version 3.6.1, http://www.R-project.org). Univariate and multivariate Cox proportional hazard regression analyses were used to evaluate the relationship between the risk scores and OS. The area under the ROC curve (AUC) (“timeROC” package in R) was used to analyze the sensitivity and specificity of genotyping and gene signature risk scores in predicting survival rate. AUC can be used as an accuracy indicator of prognosis. All statistical P values were bilateral in all the analyses, and P < 0.05 was statistically significant. The primary prognosis endpoint was overall survival, and survival curves were estimated using the Kaplan–Meier method. The log-rank test was used to determine the significance of the difference. The “Surv-cut point” function that repeatedly tests all possible cut-off points to obtain the largest rank statistic was used to dichotomize the differential genes, and then, the largest log-rank statistic was selected to divide the patients into high and low subgroups to reduce the calculated batch effect. The Paired t-test was performed to analyze statistical significance of qRT-PCR data.

## Results

### Overall Design of This Study

**Figure 1:**
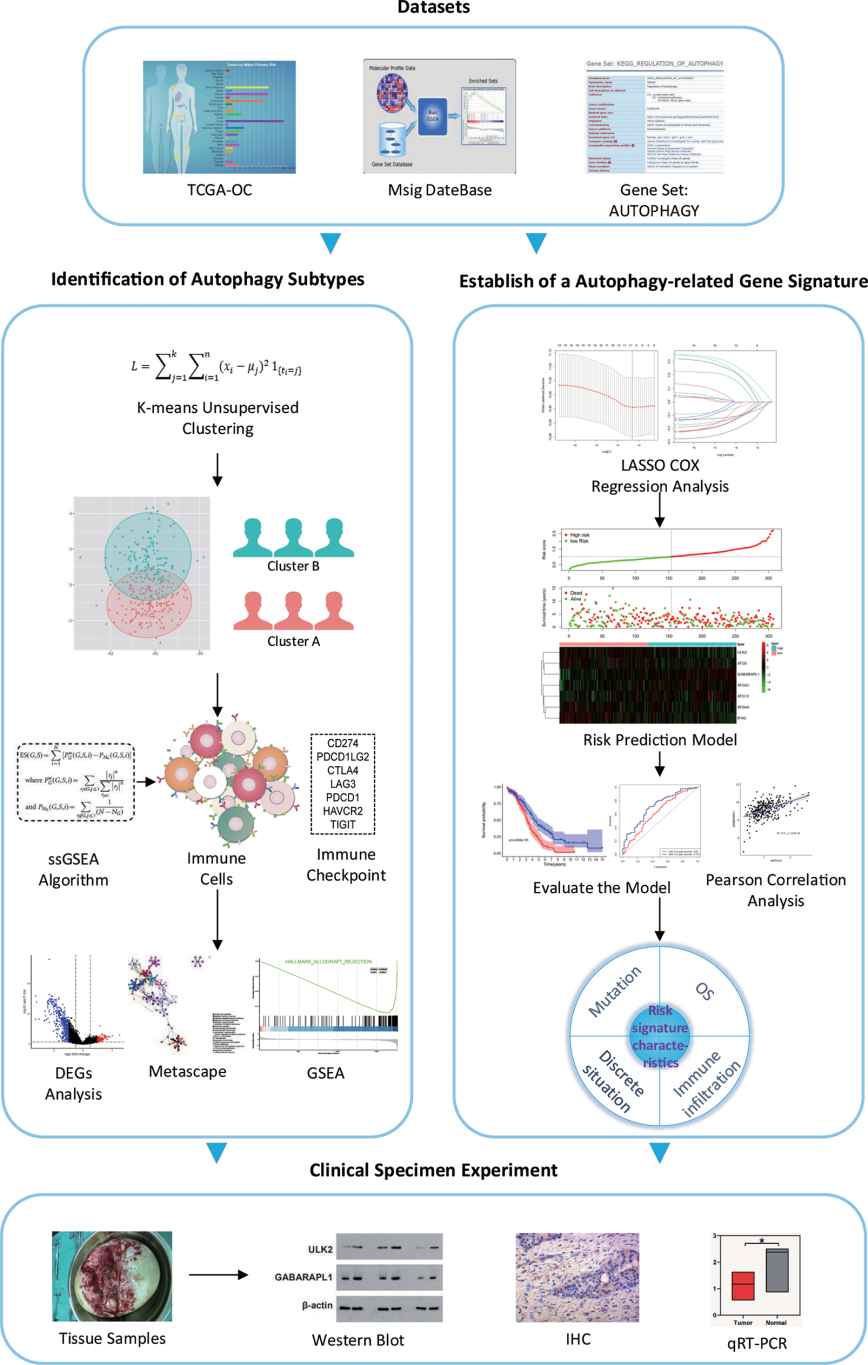
Flow chart of the study. *P < 0.05.

We have developed a flow chart to systematically describe our research (**Figure 1**). The clinical data and corresponding gene expression profiles of OC patients were downloaded from the TCGA database. One patient without prognostic data was excluded. The ARG sets were downloaded from the MsigDB for follow-up analysis. The subtypes of OC were classified by k-means unsupervised clustering and were divided into cluster A and cluster B. Then, the biological functions, metabolic pathways and signal transduction pathways with significant enrichment of differential genes were analyzed by the Metascape database, and the signaling pathways enriched in cluster A and cluster B were analyzed by GSEA. Seven ARGs were obtained through LASSO Cox regression analysis, and the risk prediction model of these ARGs was established and evaluated. The correlation between risk genes and immune cells was analyzed, and the expression of the ARGs in OC tissues and paracancerous tissues was verified by experiments.

### Identification of Two Subtypes of OC

**Figure 2:**
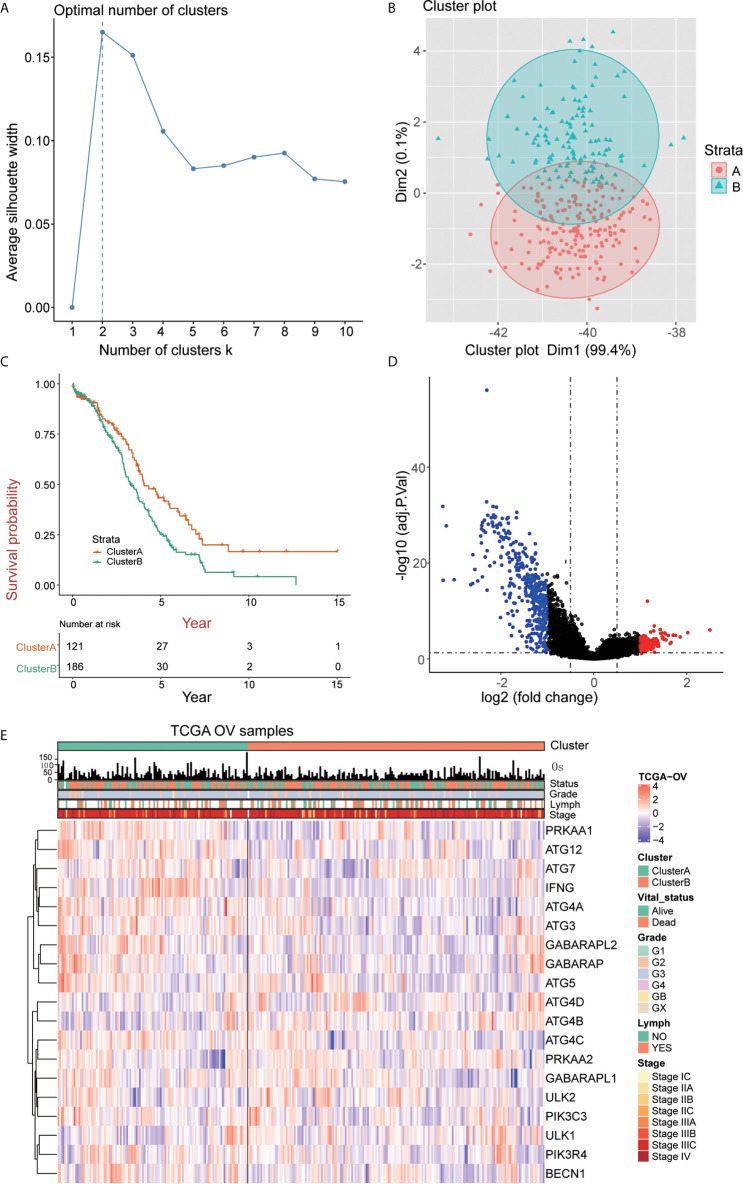
Identification of OC subtypes using k-means consensus clustering of the metadata set. **(A, B)** k-means clustering of OC based on the estimated abundance of 19 ARGs. **(C)** Kaplan‐Meier curves of the overall survival of the different gene subtypes. **(D)** Volcano plot of the identified differentially expressed genes (| logFC> 1, adjusted P <0.05). Red and blue dots indicate upregulated (N=108) and downregulated genes (N=427) in OC, respectively. **(E)** The heatmap shows the associations between the ARGs and the clinical characteristics (age, grade, lymphovascular invasion indicator, primary site and clinical stage) in the TCGA database. (Lymph = lymphovascular invasion indicator).

The 307 patients with OC clustered by k-means based on the mRNA expression of 19 ARGs. It was found that 2 was the optimal and stable number of clusters (**Figures 2A, B**). Most of the patients were enriched in cluster B, which correlated with a poor prognosis of OC (**Figure 2C**). Cluster A tended to have better survival than cluster B. 535 differentially expressed genes (DEGs) between cluster A and cluster B were identified, as shown by a volcano map these genes included 108 upregulated genes (log2 fold change ≥ 1) and 427 downregulated genes (log2 fold change ≤ -1) (**Figure 2D**). We analyzed the association among the 19 ARGs from the TCGA RNA-seq data and the clinicopathological features, including grade, stage, lymph node metastasis, survival status and overall survival time, of 307 OC patients (**Figure 2E**).

### Estimation of Cell Infiltration Into the TME

**Figure 3:**
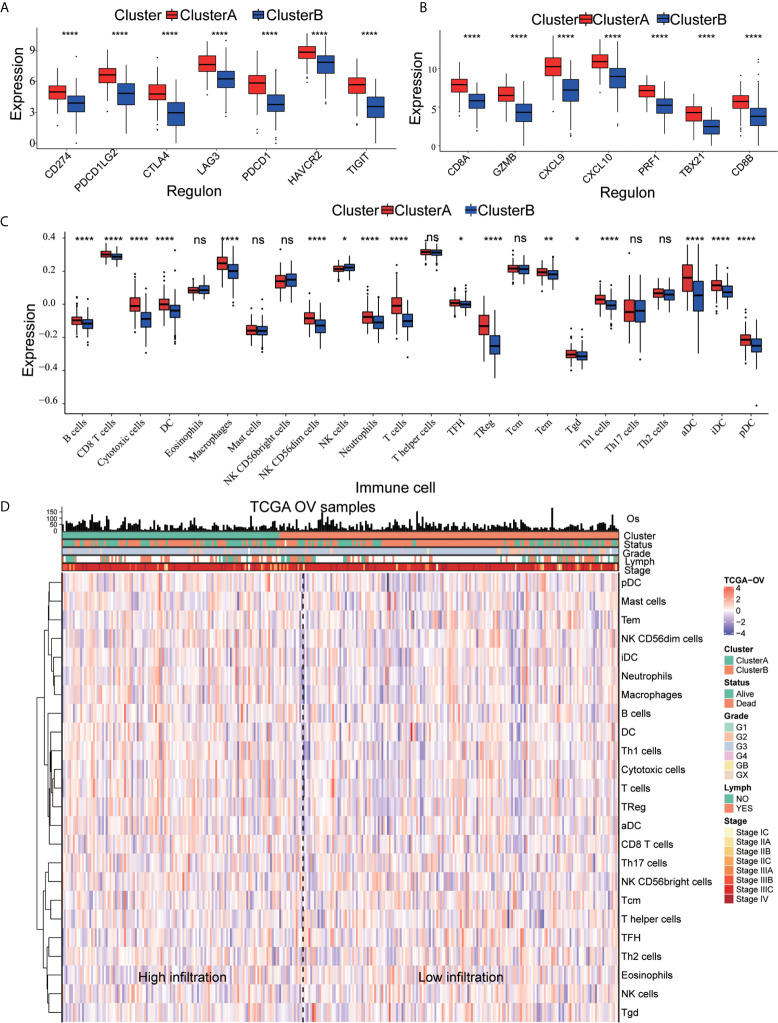
Immune characteristics of cluster A and cluster B **(A)** Expression pattern of immune checkpoints in cluster A and cluster B (****p<0.0001, **p<0.01; *p<0.05; ns<1). **(B)** Expression pattern of CD8+ T signature genes in cluster A and cluster B (****p<0.0001, ***p<0.001; **p<0.01; *p<0.05; ns<1). **(C)** The ssGSEA method quantifies the level of infiltration of the 24 immune cells in the TIM. Differences in the levels of infiltration of the 24 immune cells in cluster A and cluster B (****p<0.0001, ***p<0.001; ns<1). **(D)** The composite heat map shows the relationship between the risk score, age, grade, lymphovascular invasion indicator, primary site and clinical stage and infiltration of the 24 immune cells.

We quantified the level of immune cell infiltration to evaluate the immune landscape of cluster A and cluster B. First, we explored the relationship among cluster A, cluster B and gene expression of seven common immune checkpoints, including CD274, PDCD1LG2, CTLA4, LAG3, PDCD1, HAVCR2 and TIGIT. These genes were selected based on drug inhibitors that are currently in clinical trials or have been specifically approved in different tumor types. The results showed that the overall expression of the common immune checkpoints in cluster A was significantly higher than that in cluster B (**Figure 3A**). Next, we analyzed the difference in the expression of common CD8^+^ T marker genes (CD8A, GZMB, CXCL9, CXCL10, PRF1, TBX21, and CD8B) in cluster A and cluster B and found that the expression of CD8^+^ T marker genes in cluster A was significantly higher than that in cluster B (**Figure 3B**). In addition, ssGSEA was used to identify the abundance of tumor-infiltrating immune cells in cluster A and cluster B. We found that the levels of B cells, T cells, Treg cells, TFH cells, macrophages, aDCs, iDCs, CD56dim cells, Th1 cells, and Tgd cells in cluster A were significantly higher than those in cluster B, while the level of infiltrating NK cells in cluster A was significantly lower than that in cluster B (**Figure 3C**). We quantified 24 kinds of immune cells, such as B cells, T cells, NK cells and macrophages, and drew a heatmap that included the relationship among age, stage, grade, lymphatic metastasis and immune infiltration. Through analyzing the heatmap, we observed that cluster A showed high immune infiltration, while cluster B, on the contrary, showed low immune infiltration (**Figure 3D**). This result indicates that the immune response of cluster A is active, while that of cluster B is suppressed; thus, we speculate that OC patients in cluster A may have a better response to immunotherapy.

### Identification of the Involved Signaling Pathways

**Figure 4:**
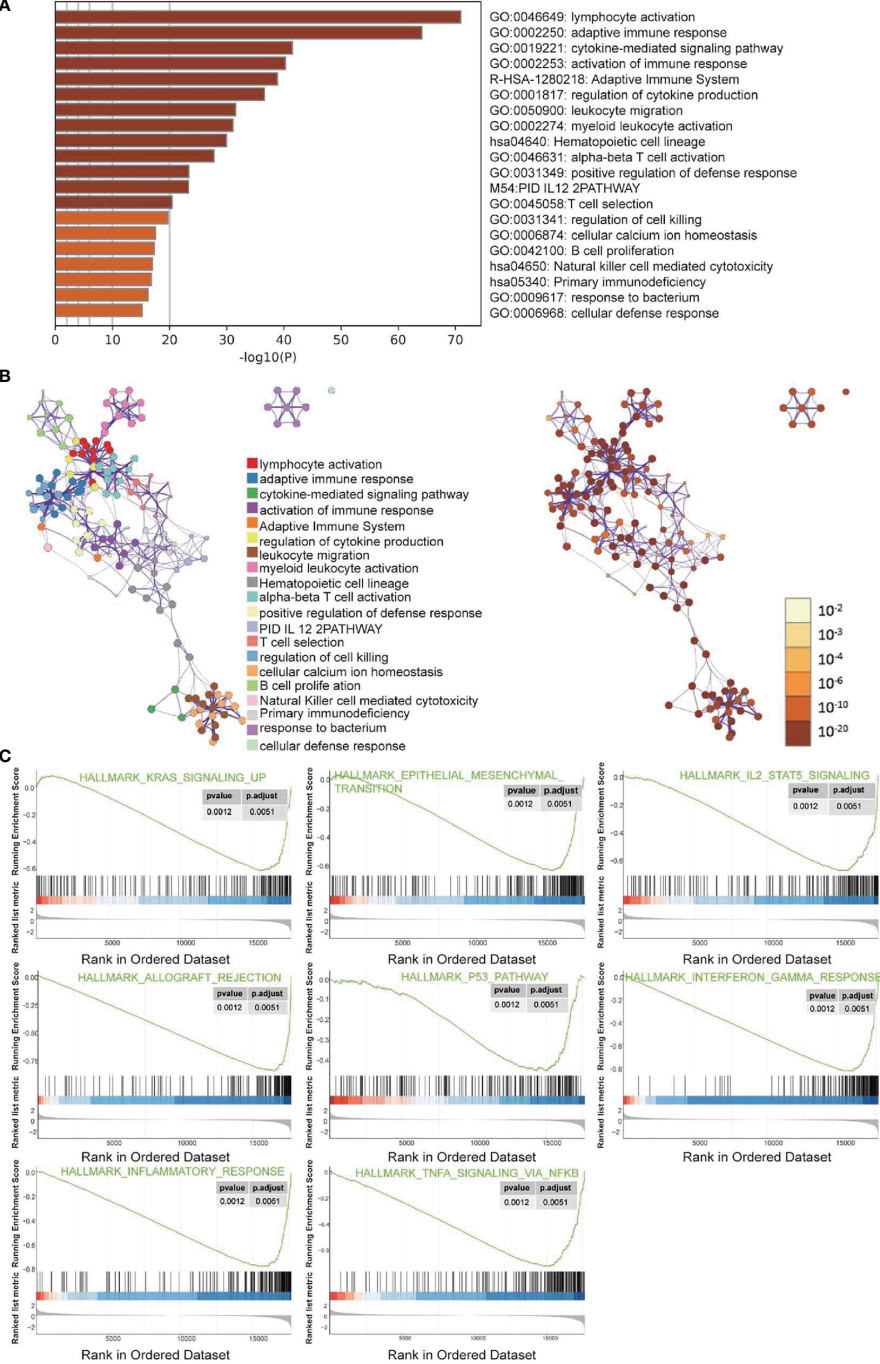
Signal pathway enrichment analysis was performed on the differentially expressed mRNAs in cluster **(A)** and cluster **(B)** For enrichment analysis in the Metascape database, each term is represented by a circular node, and its size is proportional to the number of input genes in the term. Nodes of the same color belong to the same cluster. Terms with similarity scores > 0.3 are linked by edges, and nodes in the same condensed network are colored with p-values. **(A, B)** The functions of 19 ARGs were mainly enriched in lymphocyte activation, adaptive immune response and cytokine-mediated signaling pathway. **(C)** Partial display of the GSEA analysis results.

DEGs were analyzed using Metascape to better understand their functional and pathway enrichment. First, all the statistical enrichment items were determined, the cumulative hypergeometric p-value and enrichment factor were calculated, and filtering was performed. Then, the remaining important terms were clustered into a tree in a hierarchical structure according to the Kappa statistical similarity between its gene members. The 0.3 kappa score was then used as a threshold to coerce the tree into term clusters. Next, we selected a subset of representative terms from this cluster and converted it into a network layout. The nodes of the same enrichment network are colored according to their p-values (**Figures 4A, B**). From the bar graphs and network graphs, we found that the terms related to immune function were the most abundant; among these terms, lymphocyte activation, adaptive immune response and cytokine-mediated signaling pathway were the 3 terms with the highest significance levels. GSEA was used to execute gene ontology functioinf and pathway enrichment analysis. GSEA analysis showed that signaling pathways such as interferon gamma, interferon alpha, IL-6 JAK STAT3, Inflammatory, P53 pathway, and hypoxia were significantly enriched in the cluster A group (**Figure 4C** and **Table 1**). Most of these pathways have been confirmed to be related to immunotherapy (25, 26), suggesting that ARGs may affect tumor immune regulation and providing a certain experimental direction for further research on immunotherapy in the future.

**Table 1:** GSEA analysis of the differential genes in cluster A and cluster B groups.

Description	setSize	enrichmentScore	NES	pvalue	p.adjust	qvalues
HALLMARK_KRAS_SIGNALING_UP	193	-0.62895	-2.05197	0.001188	0.005142	0.002815
HALLMARK_EPITHELIAL_MESENCHYMAL_TRANSITION	194	-0.64791	-2.11158	0.001189	0.005142	0.002815
HALLMARK_IL2_STAT5_SIGNALING	194	-0.68451	-2.23087	0.001189	0.005142	0.002815
HALLMARK_ALLOGRAFT_REJECTION	195	-0.85979	-2.8003	0.001192	0.005142	0.002815
HALLMARK_COMPLEMENT	195	-0.70364	-2.29173	0.001192	0.005142	0.002815
HALLMARK_P53_PATHWAY	191	-0.48831	-1.59174	0.001195	0.005142	0.002815
HALLMARK_INTERFERON_GAMMA_RESPONSE	196	-0.83292	-2.71133	0.001196	0.005142	0.002815
HALLMARK_INFLAMMATORY_RESPONSE	197	-0.78473	-2.55049	0.0012	0.005142	0.002815
HALLMARK_TNFA_SIGNALING_VIA_NFKB	197	-0.73492	-2.38861	0.0012	0.005142	0.002815
HALLMARK_APOPTOSIS	158	-0.60641	-1.93204	0.001239	0.005142	0.002815
HALLMARK_COAGULATION	136	-0.57384	-1.79503	0.001267	0.005142	0.002815
HALLMARK_INTERFERON_ALPHA_RESPONSE	92	-0.82712	-2.47088	0.001332	0.005142	0.002815
HALLMARK_IL6_JAK_STAT3_SIGNALING	87	-0.7752	-2.30111	0.001337	0.005142	0.002815
HALLMARK_MTORC1_SIGNALING	192	-0.45225	-1.47348	0.00479	0.017109	0.009365
HALLMARK_ADIPOGENESIS	189	-0.42693	-1.39053	0.014337	0.04779	0.026159
HALLMARK_HYPOXIA	190	-0.42317	-1.37802	0.01555	0.048594	0.026599

### Autophagy-Related Prognosis Classifier and Clinicopathological Characteristics of OC

**Figure 5:**
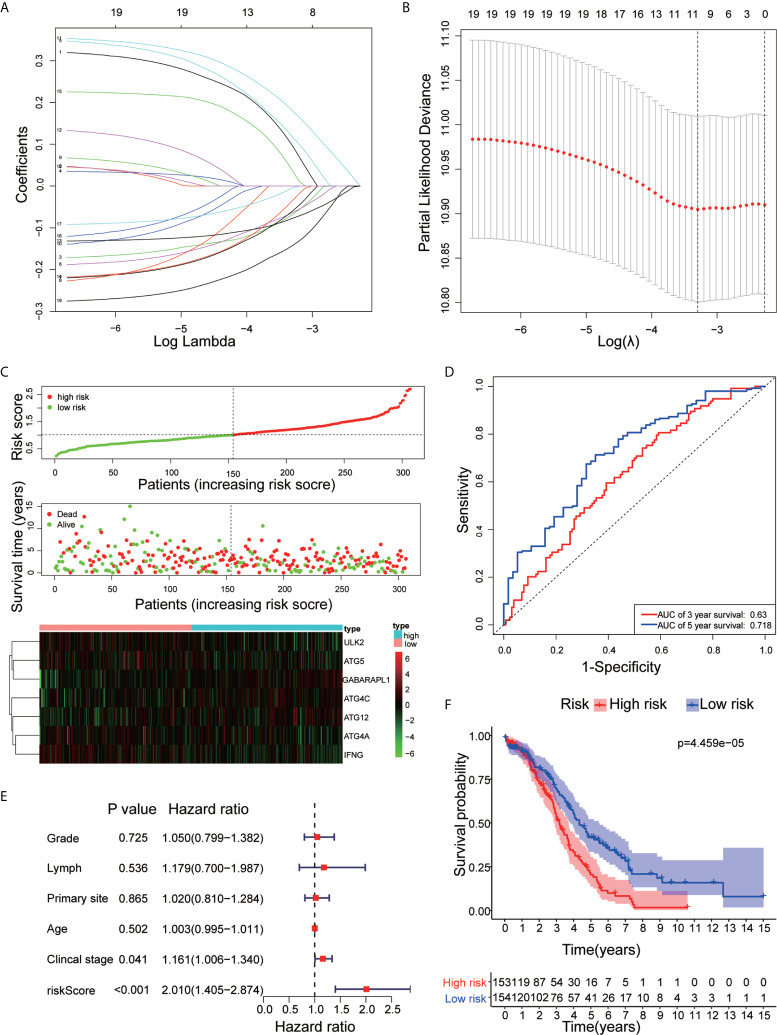
Construction of the ARG prognostic classifier. **(A, B)** Determination of the number of factors by LASSO analysis. **(C)** The distribution of risk score, survival duration and status of patients and a heatmap of the ARGs in the classifier. **(D)** The signature was evaluated by using the sensitivity and specificity of the ROC curve. **(E)** Univariate Cox proportional hazards regression analysis and the correlation between the risk score, age, grade, lymphovascular invasion indicator, primary site and clinical stage. **(F)** Kaplan-Meier analysis of TCGA OC patients stratified by median risk score.

We performed LASSO Cox regression analysis on 19 ARGs and further obtained 7 ARGs (ATG12, ATG4A, ATG4C, ATG5, GABARAPL1, IFNG, and ULK2) (**Figures 5A, B**). A risk score to predict the prognostic value of these genes was calculated, and patients with OC were separated into the low-risk or high-risk groups. We found that with the risk score increased, the number of deaths increased (**Figure 5C**). The ROC curve shows that the classifier has strong predictive ability, with an AUC of 0.63 in 3 years and 0.718 in 5 years (**Figure 5D**). Univariate Cox regression showed that the risk core was a risk factor for DFS (HR>1, p<0.05), and it had a better prognostic effect than other clinical indicators (**Figure 5E**). Kaplan-Meier analysis indicated that high-risk patients had significantly worse overall survival than low-risk patients (**Figure 5F**). This result indicates that the risk model may serve as a promising indicator for evaluating the prognosis of OC patients and may be a powerful prognostic indicator.

### Use of 7 ARGs in Survival Analysis of OC

**Figure 6:**
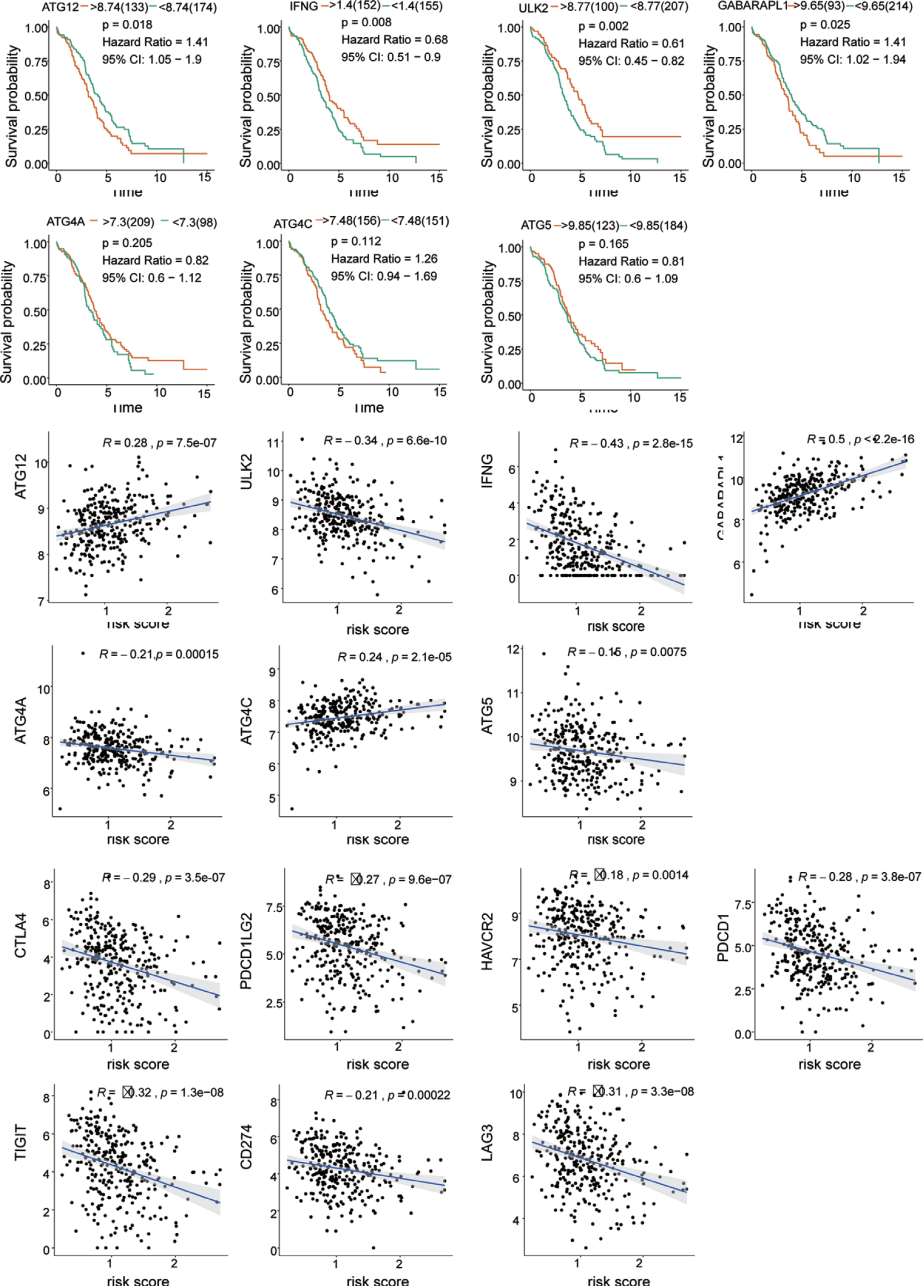
Correlation of risk score with 7 ARG signatures and important immune checkpoints. **(A)** Kaplan-Meier survival analysis of the 7 ARGs in the TCGA-OV cohort. **(B)** Correlation between risk score and 7 ARGs. **(C)** Correlation between risk score and immune checkpoints.

We evaluated the prognostic values of the 7 ARGs in OC. As shown in **Figure 6A**, 4 of the 7 ARGs played significant prognostic roles in OC: ATG12 [P= 0.018; Hazard Ratio (95% CI)=1.41(1.05-1.9)]; GABARAPL1 [P=0.025; Hazard Ratio (95% CI)=1.41(1.02-1.94)]; ULK2 [P=0.002; Hazard Ratio (95% CI) =0.61 (0.45-0.82)]; and IFNG [P=0.008; Hazard Ratio (95% CI)=0.68(0.51-0.9)]. However, ATG4A, ATG4C and ATG5 were not statistically significant. The coefficients of these genes are shown in **Table 2**. By analyzing the relationship between the 7 ARGs and OS, it was found that the group showing higher expression of the ATG12 and GABARAPL1 mRNAs had a lower survival time (P<0.05). The group showing higher expression of the ULK2 and IFNG had a significantly longer survival time than the group showing lower expression (P<0.05). We found that among the 7 genes, IFNG and ULK2 were protective factors (HR<1), while ATG12 and GABARAPL1 were risk factors (HR > 1).

**Table 2:** Univariate and multivariate COX regression analysis results of 7 immune genes.

Gene_symbol	Enxemble ID	Univariate Cox regression analysis				Multivariate Cox regression analysis			
		HR	HR.95L	HR.95H	pvalue	HR	HR.95L	HR.95H	pvalue
ATG12	ENSG00000145782.12	1.180578	0.875524	1.59192	0.27642	1.329719	0.964576	1.833089	0.081899
ATG4A	ENSG00000101844.17	0.864882	0.687113	1.088644	0.216272	0.81017	0.634949	1.033746	0.090447
ATG4C	ENSG00000125703.14	1.221694	0.90893	1.642081	0.184473	1.404834	1.028324	1.9192	0.032726
ATG5	ENSG00000057663.12	0.896569	0.711928	1.129098	0.353424	0.822211	0.647758	1.043649	0.107651
GABARAPL1	ENSG00000139112.10	1.237474	1.039371	1.473335	0.016675	1.364937	1.13734	1.638079	0.00083
IFNG	ENSG00000111537.4	0.891084	0.801327	0.990896	0.03327	0.875218	0.784645	0.976245	0.016788
ULK2	ENSG00000083290.19	0.835348	0.684796	1.018998	0.076005	0.766131	0.625682	0.938107	0.009928

### Predicting the Effectiveness of ARGs and Correlation With Our Risk Score

The results of Pearson’s correlation analysis indicated that our risk score was significantly correlated with the mRNA expression level of ARGs, namely, ATG12 (R =0.26, p =7.5e-07), GABARAPL1 (R =0.5, p <2.2e-16), ULK2 (R =0.34, p =6.6e-10), IFNG (R =-0.43, p =2.8e-15), ATG4A (R =-0.21, p =0.00015), ATG4C (R =0.24, p =2.1e-05) and ATG5 (R =-0.15, p =0.0075). It was shown that GABARAPL1 had the highest degree of correlation with the risk score. Additionally, the risk score was also markedly related to the mRNA expression level of the immune checkpoints, which are HAVCR2 (R =-0.18, p =0.0014), LAG3 (R=-0.31, p=3.3e-08), PDCD1LG2 (R=-0.27, p=9.6e-07), PDCD1 (R=-0.28, p =3.8e-07), CD274 (R=-0.21, p =0.0002) and TIGIT (R =-0.32, p=1.3e-08). The results are visualized in **Figures 6B, C**. The results reveal that patients might respond to therapies that target immune checkpoint inhibitors.

### Prognostic Signature Is Related to the TIM

**Figure 7:**
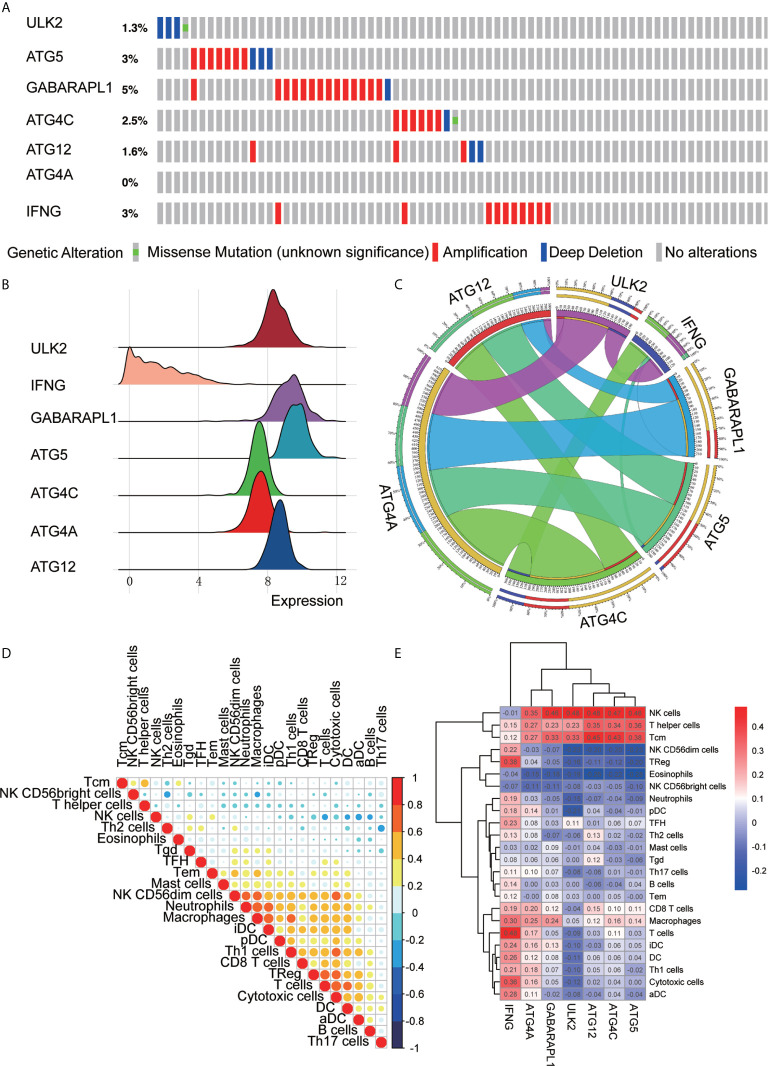
ARG signature characteristics. **(A)** The waterfall plot of OC mutations established by 7 ARGs in the TCGA-OV cohort. **(B)** The height of the mountain represents the discrete situation between a set of data. The steeper the mountain is, the more concentrated the distribution of this group of data is, and there are few discrete values between the data; the gentler the mountain is, the more dispersed the group of data is. **(C)** Circos plot shows the correlations between the 7 ARGs. **(D)** Correlations between the 24 immune cells. **(E)** Heat map of the correlation between the 7 ARGs and the 24 immune cells.

The mutations in the 7 ARGs in the TCGA-OV cohort were downloaded from the cbioportal website (https://www.cbioportal.org/). The gene of highest mutation frequency is GABARAPL1, reaching 5%, of which the main type of mutation was amplification mutations. Following genes were ATG5, IFNG, and ATG4C, which were 3, 3, and 2.5%, respectively (**Figure 7A**). We also found that the expression of IFNG in OC was more scattered and lower, while the expression of ATG12, ATG4A, ATG4C, ATG5, GABARAPL1, and ULK2 was relatively concentrated and higher (**Figure 7B**). A correlation analysis of the 7 ARGs was performed to obtain Circos plots (**Figure 7C**). We explored the correlation between 7 ARGs and immune cells (**Table 3**).

**Table 3:** Correlation between 7 ARGs and immune cells.

Immune cells	Gene markers	IFNG		ATG12		ATG4A		ATG4C		ATG5		GABARAPL1		ULK2	
		Cor	P	Cor	P	Cor	P	Cor	P	Cor	P	Cor	P	Cor	P
CD8+Tcell	CD8A	0.783	****	0.125	*	0.308	****	0.145	*	0.005	NS	0.140	*	-0.064	NS
	CD8B	0.624	****	0.062	NS	0.349	****	0.164	**	0.049	NS	0.092	NS	-0.051	NS
T	CD3D	0.771	****	0.144	*	0.277	****	0.096	NS	-0.014	NS	0.049	NS	-0.075	NS
	CD3E	0.755	****	0.102	NS	0.280	****	0.075	NS	-0.103	NS	0.067	NS	-0.062	NS
	CD2	0.762	****	0.134	*	0.293	****	0.097	NS	-0.055	NS	0.048	NS	-0.044	NS
Bcell	CD19	0.302	****	0.014	NS	0.122	*	0.121	*	0.034	NS	-0.070	NS	0.039	NS
	CD79A	0.573	****	-0.035	NS	0.195	***	0.038	NS	0.050	NS	0.059	NS	-0.055	NS
Monocyte	CD86	0.559	****	0.101	NS	0.305	****	0.226	****	-0.072	NS	0.078	NS	0.014	NS
	CSF1R	0.375	****	0.019	NS	0.187	***	0.144	*	-0.196	***	0.090	NS	0.042	NS
TAM	CCL2	0.401	****	0.231	****	0.197	***	0.079	NS	-0.074	NS	0.048	NS	-0.016	NS
	CD68	0.521	****	0.116	*	0.337	****	0.177	**	-0.081	NS	0.053	NS	-0.024	NS
	IL10	0.314	****	0.235	****	0.236	****	0.137	*	0.037	NS	0.080	NS	0.007	NS
M 1Macrophage	NOS2	-0.083	NS	-0.006	NS	0.049	NS	-0.048	NS	0.029	NS	0.143	*	0.180	**
	IRF5	0.323	****	0.047	NS	0.199	***	-0.009	NS	-0.094	NS	0.010	NS	0.117	*
	PTGS2	0.082	NS	0.157	**	0.092	NS	0.069	NS	-0.006	NS	0.165	**	0.052	NS
M2 Macrophage	CD163	0.399	****	0.094	NS	0.248	****	0.143	*	-0.165	**	0.091	NS	0.025	NS
	VSIG4	0.345	****	0.161	**	0.312	****	0.183	**	-0.063	NS	0.096	NS	-0.018	NS
	MS4A4A	0.468	****	0.156	**	0.337	****	0.185	**	-0.042	NS	0.120	*	0.031	NS
Neutrophils	ITGAM	0.415	****	0.032	NS	0.195	***	0.146	*	-0.199	***	0.047	NS	0.033	NS
	CCR7	0.591	****	0.072	NS	0.234	****	0.084	NS	-0.070	NS	0.071	NS	0.036	NS
Natural killer cell	KIR2DL1	0.263	****	0.063	NS	0.021	NS	-0.028	NS	-0.088	NS	0.087	NS	0.003	NS
	KIR2DL3	0.270	****	0.149	**	0.052	NS	-0.023	NS	-0.045	NS	0.018	NS	-0.017	NS
	KIR2DL4	0.586	****	0.162	**	0.154	**	0.027	NS	-0.027	NS	-0.028	NS	-0.026	NS
	KIR3DL1	0.364	****	0.081	NS	0.011	NS	0.008	NS	0.008	NS	-0.026	NS	0.032	NS
	KIR3DL2	0.335	****	0.074	NS	0.095	NS	0.030	NS	-0.058	NS	0.027	NS	0.061	NS
	KIR3DL3	0.129	*	0.049	NS	-0.001	NS	-0.020	NS	-0.007	NS	0.050	NS	0.010	NS
	KIR2DS4	0.277	****	0.099	NS	0.073	NS	-0.023	NS	-0.067	NS	0.015	NS	0.030	NS
Dendritic cell	HLA-DPB1	0.576	****	0.090	NS	0.252	****	0.146	*	-0.034	NS	-0.011	NS	-0.134	*
	HLA-DQB1	0.432	****	0.062	NS	0.278	****	0.121	*	-0.025	NS	0.062	NS	-0.117	*
	HLA-DRA	0.540	****	0.139	*	0.300	****	0.190	***	-0.012	NS	0.015	NS	-0.129	*
	HLA-DPA1	0.560	****	0.085	NS	0.237	****	0.172	**	-0.024	NS	0.021	NS	-0.094	NS
	CD1C	0.313	****	0.053	NS	0.039	NS	0.020	NS	-0.072	NS	0.075	NS	0.030	NS
	NRP1	0.172	****	0.091	NS	0.158	**	0.035	NS	-0.130	*	0.060	NS	0.071	NS
	ITGAX	0.509	****	0.069	NS	0.205	***	0.091	NS	-0.220	****	0.022	NS	0.026	NS
Th1	TBX21	0.777	****	0.076	NS	0.243	****	0.074	NS	-0.120	*	0.029	NS	-0.022	NS
	STAT4	0.668	****	0.120	*	0.250	****	0.056	NS	-0.054	NS	0.095	NS	0.078	NS
	STAT1	0.455	****	-0.050	NS	0.165	**	0.096	NS	-0.040	NS	0.012	NS	0.062	NS
	IFNG	1.000	NS	0.113	*	0.275	****	0.061	NS	0.010	NS	-0.001	NS	-0.054	NS
	TNF	0.259	****	0.014	NS	0.098	NS	-0.049	NS	-0.161	**	-0.102	NS	-0.052	NS
Th2	GATA3	0.412	****	0.102	NS	0.115	*	-0.029	NS	-0.144	*	0.105	NS	-0.121	*
	STAT6	0.144	*	-0.091	NS	-0.026	NS	-0.050	NS	0.057	NS	0.072	NS	-0.070	NS
	STAT5A	0.202	****	-0.016	NS	0.047	NS	0.070	NS	-0.046	NS	0.096	NS	0.075	NS
	IL13	0.251	****	0.111	NS	0.059	NS	-0.081	NS	-0.044	NS	0.051	NS	0.066	NS
Tfh	BCL6	-0.043	NS	-0.074	NS	-0.016	NS	-0.086	NS	-0.229	****	-0.096	NS	-0.139	*
	IL21	0.397	****	-0.008	NS	0.165	**	0.059	NS	0.029	NS	0.016	NS	-0.083	NS
Th17	STAT3	0.187	****	-0.011	NS	0.179	**	0.045	NS	0.025	NS	0.152	**	0.060	NS
	IL17A	0.288	****	0.026	NS	0.007	NS	-0.054	NS	-0.095	NS	-0.072	NS	-0.042	NS
Treg	FOXP3	0.670	****	0.046	NS	0.253	****	0.033	NS	-0.118	*	0.009	NS	0.022	NS
	CCR8	0.427	****	-0.003	NS	0.162	**	0.101	NS	-0.020	NS	0.056	NS	0.005	NS
	STAT5B	-0.016	NS	0.001	NS	-0.086	NS	0.006	NS	-0.005	NS	0.015	NS	0.242	****
	TGFB1	0.358	****	0.076	NS	0.310	****	0.079	NS	-0.126	*	0.070	NS	0.017	NS
T cell exhaustion	PDCD1	0.725	****	0.077	NS	0.279	****	0.056	NS	-0.060	NS	0.009	NS	-0.082	NS
	CTLA4	0.737	****	0.117	*	0.267	****	0.101	NS	-0.017	NS	0.047	NS	-0.011	NS
	LAG3	0.668	****	0.079	NS	0.199	***	0.075	NS	0.018	NS	0.041	NS	0.051	NS
	HAVCR2	0.545	****	0.129	*	0.326	****	0.229	****	-0.057	NS	0.052	NS	-0.011	NS
	GZMB	0.712	****	0.211	***	0.274	****	-0.020	NS	0.003	NS	-0.010	NS	-0.162	**
	ATG12	0.113	*	1.000	NS	0.156	**	0.100	NS	0.119	*	0.062	NS	-0.020	NS
	ATG4A	0.275	****	0.156	**	1.000	NS	0.205	***	0.135	*	0.156	**	-0.123	*
	ATG4C	0.061	NS	0.100	NS	0.205	***	1.000	NS	0.139	*	0.025	NS	0.066	NS
	ATG5	0.010	NS	0.119	*	0.135	*	0.139	*	1.000	NS	0.156	**	0.074	NS
	GABARAPL1	-0.001	NS	0.062	NS	0.156	**	0.025	NS	0.156	**	1.000	NS	0.084	NS
	ULK2	-0.054	NS	-0.020	NS	-0.123	*	0.066	NS	0.074	NS	0.084	NS	1.000	NS

*P < 0.05, **P < 0.01, ***P < 0.001, ****P < 0.0001, NS, no significance (P>0.05).

Correlation analysis was also performed to analyze the 24 immune cells. The immune cell proportions were weakly to strongly correlated. T cells and cytotoxic cells showed the strongest positive correlation; macrophages also indicated a strongly positive correlation with iDCs. Eosinophils and Th17 cells showed a moderate negative correlation (**Figure 7D**). Additionally, we analyzed the correlation between 7 immune genes and immune cells and found that ATG5, ATG4C, ATG12, ULK2, and GABARAPL1 were positively correlated with NK cells, T helper cells, Tcm cells and other immune infiltrating cells and negatively correlated with NK CD56dim cells, eosinophils and NK CD56bright cells. IFNG was positively correlated with B cells, CD8 T cells, macrophages, iDCs, DCs, Th1 cells, cytotoxic cells, aDCs, Treg cells, and TFH cells but negatively correlated with NK cells and NK CD56dim cells. ATG4A was positively correlated with macrophages, T cells, pDCs, Tcm cells, NK cells, and T helper cells and negatively correlated with eosinophils and NK CD56dim cells (**Figure 7E**).

### Validation of the Gene Signature in Clinical Tissue Samples

**Figure 8:**
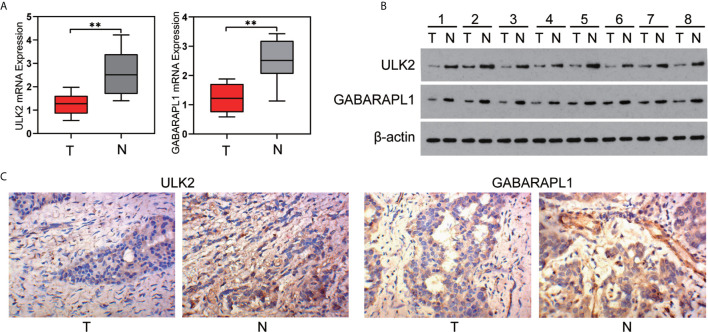
Expression of ULK2 and GABARAPL1 in OC. **(A, B)** ULK2 and GABARAPL1 expression decreased in OC tissues, compared to the paired paracancerous tissues specimens from 8 patients, determined by qRT-PCR analysis and western blot analysis. **(C)** Representative immunohistochemical images of ULK2 and GABARAPL1 expression in OC tissues and paracancerous tissues analyzed by IHC (400×). T, tumor; N, paracancerous tissues. **P < 0.01.

To confirm the reliability of the identified gene signature, we examined the ULK2 and GABARAPL1 expression levels by qRT-PCR, WB, and IHC using 8 pairs of OC tumor tissues and paracancerous tissues. The results showed that the ULK2 and GABARAPL1 mRNA in the tumor tissues were significantly downregulated compared with those in the paracancerous tissues (p<0.05) (**Figure 8A**). Moreover, the protein level of ULK2 and GABARAPL1 were detected using IHC in 8 OC clinical tissues that underwent cytoreductive surgery. The IHC analysis showed that ULK2 and GABARAPL1 were lowly expressed in the OC tissues (**Figures 8B, C**).

## Discussion

The early symptoms of patients with OC can’t be easily detectable. Due to difficulty in diagnosis at early stage, tumor recurrence, chemoresistance, and poor prognosis, improving clinical treatment is very challenging. Therefore, it is urgent to identify reliable biomarkers for early prognosis to facilitate the diagnosis and treatment of OC. Induction of autophagy has shown promise in the management of a wide range of illnesses, including neurodegenerative disorders, cardiovascular diseases, and rheumatic diseases (27–29). Accumulating evidence suggests that autophagy plays important roles in tumor chemotherapy and radiotherapy. In addition, autophagy affects TIM and tumor immunotherapy. Proper regulation of autophagy could enhance the immune response, leading to immunotherapy potentiation.

Recently, a medical breakthrough was made in the field of cancer immunotherapy. However, how the immune system uses immune cells to eradicate tumors in a complex environment remains to be further investigated. Unfortunately, only a small proportion of cancer patients and only some cancers currently respond to immunotherapy (30). How to make more patients benefit from personalized immunotherapy and improve the efficacy of immunotherapy is the focus of tumor immunology. Immune cells play a protective role in the tumor microenvironment, which can lead to immune checkpoint inhibition and immune cell infiltration (31). Therapeutic strategies that target the immune microenvironment, such as immunosuppressive cells and immunosuppressive factors, can effectively prevent tumor cells from escaping immune surveillance. Therapies that block immune checkpoints show longer-lasting responses than traditional chemotherapy and have been approved by the FDA for the treatment of multiple cancer types, such as melanoma, nonsquamous cell carcinoma, OC, etc. (32–34). However, therapies that block immune checkpoints have low response rates in approximately 10%-30% cancers, which may be related to tumor mutational burden, PD-L1 expression level, IFN signaling and MHC-I loss. However, the current understanding of the low response rate of immunotherapy is still limited, and the regulatory mechanism is not clear.

Targeting the tumor microenvironment by immunotherapy may be a promising therapeutic strategy. Autophagy regulates innate and adaptive immune responses by regulating immune components, such as NK cells, T lymphocytes, B cells and DC cells (35). It was reported that autophagy could promote antigen presentation by MHCII and MHCI molecules on APCs. Autophagy provides an important antigen source for the loading of MHCII, thus activating antigen-specific CD8^+^ T cells (36). In addition, autophagy not only regulates cytokines to affect immunity but also increases the response of MHCI molecules to IFN-γ to enhance cross-presentation (37). The defection of autophagy causes the accumulation of intracellular fat droplets, promotes the release of linoleic acid, and leads to the exhaustion of liver CD411 cells, thereby inducing an immunosuppressive tumor microenvironment and promoting tumor progression (38, 39). Antibody-mediated responses to both T cell-dependent and T cell-independent antigens also require autophagy. Otherwise, these responses will cause endoplasmic reticulum stress and T cell death. Autophagy-related gene suppression in human pancreatic ductal adenocarcinoma 8988T cells can promote the expression of PD-L1, which is conducive to the establishment of an immunosuppressive tumor microenvironment (40).

There have been no comprehensive and systematic studies on the relationship among autophagy, the immune microenvironment and immunotherapy in OC. This study aims to identify suitable immunotherapy targets and effective prognostic biomarkers and to predict the response and prognosis of OC. Using k-means clustering, we successfully divided OC samples into two groups, cluster A and cluster B. Significant differences were found between cluster A and cluster B. Cluster A had a better prognosis for OS and had a higher immune infiltration level than cluster B. Next, we use Metascape to analyze the differential mRNA (FDR<0.05) of cluster A and cluster B for signal pathway enrichment analysis. Lymphocyte activation, adaptive immune response and cytokine-mediated signaling pathway were the top three signaling pathways, and all of these pathways were closely related to the function of the immune system. We then used GESA for pathway enrichment and found that IFN-γ, IFN-α, IL-6 JAK STAT3, inflammatory, P53 pathway, and hypoxia were significantly enriched in cluster A. Most of these pathways are closely related to tumorigenesis and the TME. For example, interferon plays a vital role in the immune response and has been studied in a variety of malignant tumors (41, 42). IFN-γ signal transduction can promote Treg function in autoimmunity, and activation of the IFN-α signaling pathway leads to a more effective antiviral response and enhanced antitumor immunity (43). The JAK/STAT3 pathway is aberrantly activated in various cancers, including OC, and is involved in functional regulation of the tumor microenvironment (44). Inflammatory factors could induce immune suppression and mediate immune escape (45). The P53 tumor suppressor pathway plays a key role in tumor immunology and the homeostatic regulation of immune responses (46). Our classification is of great significance in guiding clinical work. Patients in cluster A have a better prognosis and significantly higher immune infiltration than those in cluster B, and the enriched signaling pathways are mainly related to the TIM. Thus, cluster A may obtain better clinical outcomes with immunotherapy and have a certain clinical reference value for OC.

Gene markers are widely used in modern clinical diagnosis. Traditional markers, such as CA125, CA199 and alpha-fetoprotein (AFP), are frequently used to diagnose OC, gastric cancer and liver cancer, predicting prognosis, and monitoring postoperative recurrence, respectively (47–49). Additional searches for more sensitive and specific markers are needed to improve clinical diagnosis and treatment. We established a 7 ARG prognostic risk signature by LASSO Cox regression and then divided patients into high-risk and low-risk groups. ROC curve and Kaplan-Meier survival analyses showed that the model was reliable and helpful to identify high-risk and low-risk patients. Univariate Cox regression analyses showed that the risk score was a more reliable indicator of DFS than other factors. The risk score was also closely related to risk genes and immune checkpoint molecules. Some risk genes have been confirmed to be closely related to the formation and development of OC. IFNG expression is related to the upregulation of XBP1, and lacking XBP1 selectively in T cells could promote antitumor immunity (50). P53 and RAS mutants regulate apoptosis and autophagy through the dysregulation of ATG12 and affect chemotherapy resistance (51). It was reported that ATG5 could promote the growth of OC cells in the peritoneal microenvironment, and inhibition of ATG5-induced autophagy sensitizes OC cells to olaparib and other PARP inhibitors (52, 53). However, GABARAPL1 and ULK2 have not been reported in OC. Experimental verification found that ULK2 and GABARAPL1 are downregulated in OC, combined with OS analysis, the results show the higher expression of ULK2 indicated a better prognosis whereas the higher expression of GABARAPL1 showed a worse prognosis in OC. ULK2 may be a protective prognostic factor. Unexpectedly, GABARAPL1 may be a risk prognostic factor, the reason may be that the sample size was too small for inferential statistics or that the characteristics of the autophagy gene itself caused this result, but the specific reasons need to be further explored. ULK2 and GABARAPL1 may be potential therapeutic targets for OC.

The successful classification of OC in this study is conducive to screening patients suitable for immunotherapy, and 7 marker genes can be used as strong biological markers for the prognosis of OC. The research, however, has some limitations. Firstly, the number of ARG sets studied is relatively small, and there is no validation set. In addition, we verified the expression of some ARGs in tissues, but the biological functions of the ARGs need further experimental research.

In summary, the autophagy-immune-based gene risk signature might be helpful to guide the clinical treatment, evaluate the prognosis and predict the efficacy of immunotherapy. ARGs might be potential markers for the prognosis of OC immunotherapy.

## Data Availability Statement

The raw data supporting the conclusions of this article will be made available by the authors, without undue reservation.

## Ethics Statement

The studies involving human participants were reviewed and approved by The IRB of Third Xiangya Hospital, Central South University. The patients/participants provided their written informed consent to participate in this study.

## Author Contributions

KC and SX designed the study. XC and HL analyzed, interpreted the data, wrote original draft. DH, ZW, RX and JY. wrote this manuscript. MX, YZ, and LG edited and revised the manuscript. All authors contributed to the article and approved the submitted version.

## Funding

This work was supported by the National Natural Science Foundation of China (81874137), the Science and Technology Innovation Program of Hunan Province (2020RC4011), the Outstanding Youth Foundation of Hunan Province (2018JJ1047), the Hunan Province Science and Technology Talent Promotion Project (2019TJ-Q10), Young Scholars of “Furong Scholar Program” in Hunan Province, and the Wisdom Accumulation and Talent Cultivation Project of the Third Xiangya Hospital of Central South University (BJ202001), Philosophy and Social Science Foundation Project of Hunan Province (19YBA349), Clinical Medical Technology Innovation Guidance Plan of Hunan Province (2020SK53607).

## Conflict of Interest

The authors declare that the research was conducted in the absence of any commercial or financial relationships that could be construed as a potential conflict of interest.

## Abbreviations

OC, Ovarian cancer; OS, Overall survival; DFS, Disease-free survival; ARGs, autophagy-related genes; TCGA, The cancer genome atlas; LASSO, The least absolute shrinkage and selection operator; GSEA, Gene set enrichment analysis; ROC, The receiver operating characteristic; SsGSEA, The single-sample gene set enrichment analysis; TIM, Tumor immune microenvironment; TME, The tumor microenvironment; ICT, The immune checkpoint therapy; CTLA-4, Cytotoxic lymphocyte antigen-4; PD-1, Programmed cell death protein 1; PD-L1, Programmed-death ligand 1; FDA, Food and drug administration; LAG3, Lymphocyte Activating 3; TIGIT, T cell Immunoreceptor with Ig and ITIM domains; BTLA, B and T lymphocyte associated; VEGF, Vascular endothelial growth factor; TLRs, Toll-like receptor; NLRs, Nucleotide oligomeric domain (NOD)-like receptor; MHCI, Major histocompatibility complex class I; MHCII, Major histocompatibility complex class II; APCs, Activate antigen presenting cells; DEGs, Differentially expressed genes; NES, Normalized Enrichment Score.

## References

[1] Bray F Ferlay J Soerjomataram I Siegel R Torre L Jemal A. Global Cancer Statistics 2018: GLOBOCAN Estimates of Incidence and Mortality Worldwide for 36 Cancers in 185 Countries. CA Cancer J Clin (2018) 68:394–424. 10.3322/caac.21492 30207593

[2] Dood RL Zhao Y Armbruster S Coleman R Tworoger S Sood A. Defining Survivorship Trajectories Across Patients With Solid Tumors: An Evidence-Based Approach. JAMA Oncol (2018) 4:1519–26. 10.1001/jamaoncol.2018.2761 PMC6248088 29860375

[3] Ray-Coquard I Pautier P Pignata S Perol D Gonzalez-Martin A Berger R. Olaparib Plus Bevacizumab as First-Line Maintenance in Ovarian Cancer. N Engl J Med (2019) 381:2416–28. 10.1056/NEJMoa1911361 31851799

[4] Wang P Jiang S Li Y Luo Q Lin J Hu L. Fabrication of Hypoxia-Responsive and Uperconversion Nanoparticles-Modified RBC Micro-Vehicles for Oxygen Delivery and Chemotherapy Enhancement. Biomater Sci (2020) 8:4595–602. 10.1039/D0BM00678E 32700684

[5] Koo M Swann R McPhail S Abel G Elliss-Brookes L Rubin G. Presenting Symptoms of Cancer and Stage At Diagnosis: Evidence From a Cross-Sectional, Population-Based Study. Lancet Oncol (2020) 21:73–9. 10.1016/S1470-2045(19)30595-9 PMC6941215 31704137

[6] Jimenez-Sanchez A Cybulska P Mager K Koplev S Cast O Couturier D. Unraveling Tumor-Immune Heterogeneity in Advanced Ovarian Cancer Uncovers Immunogenic Effect of Chemotherapy. Nat Genet (2020) 52:582–93. 10.1038/s41588-020-0630-5 PMC8353209 32483290

[7] Gul A Stewart T Mantia C Shah N Gatof E Long Y. Salvage Ipilimumab and Nivolumab in Patients With Metastatic Renal Cell Carcinoma After Prior Immune Checkpoint Inhibitors. J Clin Oncol (2020) 38:JCO1903315. 10.1200/JCO.19.03315 PMC7499610 32491962

[8] Arbyn M Weiderpass E Bruni L de Sanjose S Saraiya M Ferlay J. Estimates of Incidence and Mortality of Cervical Cancer in 2018: A Worldwide Analysis. Lancet Glob Health (2020) 8:e191–203. 10.1016/S2214-109X(19)30482-6 PMC7025157 31812369

[9] Aoki T Chong L Takata K Milne K Hav M Colombo A. Single-Cell Transcriptome Analysis Reveals Disease-Defining T-cell Subsets in the Tumor Microenvironment of Classic Hodgkin Lymphoma. Cancer Discov (2020) 10:406–21. 10.1158/2159-8290.CD-19-0680 31857391

[10] Zamarin D Burger R Sill M Powell D Lankes H Feldman M. Randomized Phase Ii Trial of Nivolumab Versus Nivolumab and Ipilimumab for Recurrent or Persistent Ovarian Cancer: An NRG Oncology Study. J Clin Oncol (2020) 38:1814–23. 10.1200/JCO.19.02059 PMC7255977 32275468

[11] Matulonis U Shapira-Frommer R Santin A Lisyanskaya A Pignata S Vergote I. Antitumor Activity and Safety of Pembrolizumab in Patients With Advanced Recurrent Ovarian Cancer: Results From the Phase II Keynote-100 Study. Ann Oncol (2019) 30:1080–7. 10.1093/annonc/mdz135 31046082

[12] Disis M Taylor M Kelly K Beck J Gordon M Moore K. Efficacy and Safety of Avelumab for Patients With Recurrent or Refractory Ovarian Cancer: Phase 1b Results From the JAVELIN Solid Tumor Trial. JAMA Oncol (2019) 5:393–401. 10.1001/jamaoncol.2018.6258 30676622 PMC6439837

[13] Liu CH Liu H Ge B. Innate Immunity in Tuberculosis: Host Defense vs Pathogen Evasion. Cell Mol Immunol (2017) 14:963–75. 10.1038/cmi.2017.88 PMC5719146 28890547

[14] Zhong Z Sanchez-Lopez E Karin M. Autophagy, Inflammation, and Immunity: A Troika Governing Cancer and Its Treatment. Cell (2016) 166:288–98. 10.1016/j.cell.2016.05.051 PMC4947210 27419869

[15] Yamamoto K Venida A Yano J Biancur D Kakiuchi M Gupta S. Autophagy Promotes Immune Evasion of Pancreatic Cancer by Degrading MHC-I. Nature (2020) 581:100–5. 10.1038/s41586-020-2229-5 PMC7296553 32376951

[16] Wahba J Natoli M Whilding L Parente-Pereira A Jung Y Zona S. Chemotherapy-Induced Apoptosis, Autophagy and Cell Cycle Arrest are Key Drivers of Synergy in Chemo-Immunotherapy of Epithelial Ovarian Cancer. Cancer Immunol Immunother (2018) 67:1753–65. 10.1007/s00262-018-2199-8 PMC6208825 30167862

[17] Lu Y Cheung Y Tang Y. Self-Adaptive Multiprototype-Based Competitive Learning Approach: A K-Means-Type Algorithm for Imbalanced Data Clustering. IEEE Trans Cybern (2021) 51:1598–612. 10.1109/TCYB.2019.2916196 31150353

[18] Bindea G Mlecnik B Tosolini M Kirilovsky A Waldner M Obenauf A. Spatiotemporal Dynamics of Intratumoral Immune Cells Reveal the Immune Landscape in Human Cancer. Immunity (2013) 39:782–95. 10.1016/j.immuni.2013.10.003 24138885

[19] Zhou Y Zhou B Pache L Chang M Khodabakhshi A Tanaseichuk O. Metascape Provides a Biologist-Oriented Resource for the Analysis of Systems-Level Datasets. Nat Commun (2019) 10:1523. 10.1038/s41467-019-09234-6 30944313 PMC6447622

[20] Castro MA de Santiago I Campbell T Vaughn C Hickey T Ross E. Regulators of Genetic Risk of Breast Cancer Identified by Integrative Network Analysis. Nat Genet (2016) 48:12–21. 10.1038/ng.3458 26618344 PMC4697365

[21] Bao Z Li M Wang J Wang C Wang H Wang W. Prognostic Value of a Nine-Gene Signature in Glioma Patients Based on mRNA Expression Profiling. CNS Neurosci Ther (2014) 20:112–8. 10.1111/cns.12171 PMC6493176 24279471

[22] Cheng W Ren X Zhang C Cai J Liu Y Han S. Bioinformatic Profiling Identifies an Immune-Related Risk Signature for Glioblastoma. Neurology (2016) 86:2226–34. 10.1212/WNL.0000000000002770 27225222

[23] Krzywinski M Schein J Birol I Connors J Gascoyne R Horsman D. Circos: An Information Aesthetic for Comparative Genomics. Genome Res (2009) 19:1639–45. 10.1101/gr.092759.109 PMC2752132 19541911

[24] Chan E Cameron D Petersen D Lyons R Baldi D Papenfuss A. Optical Mapping Reveals a Higher Level of Genomic Architecture of Chained Fusions in Cancer. Genome Res (2018) 28:726–38. 10.1101/gr.227975.117 PMC5932612 29618486

[25] Waldmann T Chen J. Disorders of the JAK/STAT Pathway in T Cell Lymphoma Pathogenesis: Implications for Immunotherapy. Annu Rev Immunol (2017) 35:533–50. 10.1146/annurev-immunol-110416-120628 PMC7974381 28182501

[26] Ni J Wang X Stojanovic A Zhang Q Wincher M Buhler L. Single-Cell RNA Sequencing of Tumor-Infiltrating Nk Cells Reveals That Inhibition of Transcription Factor HIF-1alpha Unleashes NK Cell Activity. Immunity (2020) 52:1075–1087 e8. 10.1016/j.immuni.2020.05.001 32445619

[27] Lee KI Choi S Matsuzaki T Alvarez-Garcia O Olmer M Grogan S. FOXO1 and FOXO3 Transcription Factors Have Unique Functions in Meniscus Development and Homeostasis During Aging and Osteoarthritis. Proc Natl Acad Sci U S A (2020) 117:3135–43. 10.1073/pnas.1918673117 PMC7022148 31980519

[28] Ali T Rahman S Hao Q Li W Liu Z Ali Shah F. Melatonin Prevents Neuroinflammation and Relieves Depression by Attenuating Autophagy Impairment Through FOXO3a Regulation. J Pineal Res (2020) 69:e12667. 10.1111/jpi.12667 32375205

[29] Rockel J Kapoor M. Autophagy: Controlling Cell Fate in Rheumatic Diseases. Nat Rev Rheumatol (2016) 12:517–31. 10.1038/nrrheum.2016.92 27334205

[30] Topalian S Taube J Pardoll D. Neoadjuvant Checkpoint Blockade for Cancer Immunotherapy. Science (2020) 367:eaax0182. 10.1126/science.aax0182 32001626 PMC7789854

[31] Neo S Yang Y Record J Ma R Chen X Chen Z. CD73 Immune Checkpoint Defines Regulatory NK Cells Within the Tumor Microenvironment. J Clin Invest (2020) 130:1185–98. 10.1172/JCI128895 PMC7269592 31770109

[32] Diab A Tannir N Bentebibel S Hwu P Papadimitrakopoulou V Haymaker C. Bempegaldesleukin (NKTR-214) Plus Nivolumab in Patients With Advanced Solid Tumors: Phase I Dose-Escalation Study of Safety, Efficacy, and Immune Activation (Pivot-02). Cancer Discov (2020) 10:1158–73. 10.1158/2159-8290.CD-19-1510 32439653

[33] Reck M Wehler T Orlandi F Nogami N Barone C Moro-Sibilot D. Safety and Patient-Reported Outcomes of Atezolizumab Plus Chemotherapy With or Without Bevacizumab Versus Bevacizumab Plus Chemotherapy in Non-Small-Cell Lung Cancer. J Clin Oncol (2020) 38:2530–42. 10.1200/JCO.19.03158 PMC7392741 32459597

[34] Pfisterer J Shannon C Baumann K Rau J Harter P Joly F. Bevacizumab and Platinum-Based Combinations for Recurrent Ovarian Cancer: A Randomised, Open-Label, Phase 3 Trial. Lancet Oncol (2020) 21:699–709. 10.1016/S1470-2045(20)30142-X 32305099

[35] Jaynes J Sable R Ronzetti M Bautista W Knotts Z Abisoye-Ogunniyan A. Mannose Receptor (CD206) Activation in Tumor-Associated Macrophages Enhances Adaptive and Innate Antitumor Immune Responses. Sci Transl Med (2020) 12. 10.1126/scitranslmed.aax6337 PMC7832040 32051227

[36] Jiang G Tan Y Wang H Peng L Chen H Meng X. The Relationship Between Autophagy and the Immune System and its Applications for Tumor Immunotherapy. Mol Cancer (2019) 18:17. 10.1186/s12943-019-0944-z 30678689 PMC6345046

[37] Sugawara E Kato M Kudo Y Lee W Hisada R Fujieda Y. Autophagy Promotes Citrullination of VIM (Vimentin) and its Interaction With Major Histocompatibility Complex Class II in Synovial Fibroblasts. Autophagy (2020) 16:946–55. 10.1080/15548627.2019.1664144 PMC7144877 31486697

[38] Pengo N Scolari M Oliva L Milan E Mainoldi F Raimondi A. Plasma Cells Require Autophagy for Sustainable Immunoglobulin Production. Nat Immunol (2013) 14:298–305. 10.1038/ni.2524 23354484

[39] Ma C Kesarwala A Eggert T Medina-Echeverz J Kleiner D Jin P. NAFLD Causes Selective CD4(+) T Lymphocyte Loss and Promotes Hepatocarcinogenesis. Nature (2016) 531:253–7. 10.1038/nature16969 PMC4786464 26934227

[40] Yang S Imamura Y Jenkins R Canadas I Kitajima S Aref A. Autophagy Inhibition Dysregulates TBK1 Signaling and Promotes Pancreatic Inflammation. Cancer Immunol Res (2016) 4:520–30. 10.1158/2326-6066.CIR-15-0235 PMC4891226 27068336

[41] Denarda D Marine B Alizée J Catherine R David B Priyanka A. Cooperation Between Constitutive and Inducible Chemokines Enables T Cell Engraftment and Immune Attack in Solid Tumors. Cancer Cell (2019) 35:885–900.e10. 10.1016/j.ccell.2019.05.004 31185212 PMC6961655

[42] Chen J Cao Y Markelc B Kaeppler J Vermeer J Muschel R. Type I IFN Protects Cancer Cells From CD8+ T Cell-Mediated Cytotoxicity After Radiation. J Clin Invest (2019) 129:4224–38. 10.1172/JCI127458 PMC6763250 31483286

[43] Werfel T Elion D Rahman B Hicks B Sanchez B Gonzales-Ericsson B. Treatment-Induced Tumor Cell Apoptosis and Secondary Necrosis Drive Tumor Progression in the Residual Tumor Microenvironment Through MerTK and IDO1. Cancer Res (2019) 79:171–82. 10.1158/0008-5472.CAN-18-1106 30413412

[44] Gritsina G Xiao F O’Brien S Gabbasov R Maglaty M Xu R. Targeted Blockade of JAK/STAT3 Signaling Inhibits Ovarian Carcinoma Growth. Mol Cancer Ther (2015) 14:1035–47. 10.1158/1535-7163.MCT-14-0800 PMC4394029 25646015

[45] Verweyen E Holzinger D Weinhage T Hinze C Wittkowski H Pickkers P. Synergistic Signaling of TLR and IFNalpha/beta Facilitates Escape of IL-18 Expression From Endotoxin Tolerance. Am J Respir Crit Care Med (2020) 201:526–39. 10.1164/rccm.201903-0659OC PMC7047449 31710506

[46] Barili V Fisicaro P Montanini B Acerbi G Filippi A Forleo G. Targeting p53 and Histone Methyltransferases Restores Exhausted CD8+ T Cells in HCV Infection. Nat Commun (2020) 11:604. 10.1038/s41467-019-14137-7 32001678 PMC6992697

[47] Carr BI D’Alessandro R Refolo M Iacovazzi P Lippolis C Messa C. Effects of Low Concentrations of Regorafenib and Sorafenib on Human HCC Cell AFP, Migration, Invasion, and Growth In Vitro. J Cell Physiol (2013) 228:1344–50. 10.1002/jcp.24291 PMC3582757 23169148

[48] Fayanju OL. Hiding in Plain Sight. JAMA (2019) 322:2173–4. 10.1001/jama.2019.19326 PMC7049073 31821434

[49] May T Oza A. Measurement Tool of Chemotherapy Sensitivity in Advanced Ovarian Cancer. Clin Cancer Res (2020) 26:4432–4. 10.1158/1078-0432.CCR-20-1376 32601078

[50] Song M Sandoval TA Chae CS Chopra S Tan M Rutkowski R. Ire1alpha-XBP1 Controls T Cell Function in Ovarian Cancer by Regulating Mitochondrial Activity. Nature (2018) 562:423–8. 10.1038/s41586-018-0597-x PMC6237282 30305738

[51] Zhang X Qi Z Yin H Yang G. Interaction Between p53 and Ras Signaling Controls Cisplatin Resistance *Via* HDAC4- and HIF-1alpha-mediated Regulation of Apoptosis and Autophagy. Theranostics (2019) 9:1096–114. 10.7150/thno.29673 PMC6401400 30867818

[52] Kuo CL Jiang Z Wang Y Lin T Huang W Wu F. In Vivo Selection Reveals Autophagy Promotes Adaptation of Metastatic Ovarian Cancer Cells to Abdominal Microenvironment. Cancer Sci (2019) 110:3204–14. 10.1111/cas.14162 PMC6778661 31385416

[53] Santiago-O’Farrill JM Weroha S Hou X Oberg A Heinzen E Maurer M. Poly(Adenosine Diphosphate Ribose) Polymerase Inhibitors Induce Autophagy-Mediated Drug Resistance in Ovarian Cancer Cells, Xenografts, and Patient-Derived Xenograft Models. Cancer (2020) 126:894–907. 10.1002/cncr.32600 31714594 PMC6992526

